# Anthelmintic resistance to ivermectin and moxidectin in gastrointestinal nematodes of cattle in Europe

**DOI:** 10.1016/j.ijpddr.2015.08.001

**Published:** 2015-08-18

**Authors:** Thomas Geurden, Christophe Chartier, Jane Fanke, Antonio Frangipane di Regalbono, Donato Traversa, Georg von Samson-Himmelstjerna, Janina Demeler, Hima Bindu Vanimisetti, David J. Bartram, Matthew J. Denwood

**Affiliations:** a Zoetis, Mercuriusstraat 20, 1930, Zaventem, Belgium; b LUNAM University, ONIRIS, Nantes-Atlantic College of Veterinary Medicine, Food Science and Engineering, UMR 1300 BIOEPAR, Nantes, F-44307, France; c Institute of Parasitology and Tropical Veterinary Medicine, Freie Universität Berlin, Germany; d Department of Animal Medicine, Production and Health, University of Padua, Padua, Italy; e Faculty of Veterinary Medicine, University of Teramo, 64100, Teramo, Italy; f Zoetis, 333 Portage Street, Kalamazoo, MI, 49007-4931, USA; g Zoetis, 23/25 avenue du Docteur Lannelongue, 75668, Paris Cedex 14, France; h Department of Large Animal Sciences, University of Copenhagen, Grønnegårdsvej 8, 1870, Frederiksberg C, Denmark

**Keywords:** Ivermectin, Moxidectin, Anthelmintic resistance, Cattle, Europe

## Abstract

Anthelmintic resistance has been increasingly reported in cattle worldwide over the last decade, although reports from Europe are more limited. The objective of the present study was to evaluate the efficacy of injectable formulations of ivermectin and moxidectin at 0.2 mg per kg bodyweight against naturally acquired gastro-intestinal nematodes in cattle. A total of 753 animals on 40 farms were enrolled in Germany (12 farms), the UK (10 farms), Italy (10 farms), and France (8 farms). Animals were selected based on pre-treatment faecal egg counts and were allocated to one of the two treatment groups. Each treatment group consisted of between 7 and 10 animals. A post-treatment faecal egg count was performed 14 days (±2 days) after treatment. The observed percentage reduction was calculated for each treatment group based on the arithmetic mean faecal egg count before and after treatment. The resistance status was evaluated based on the reduction in arithmetic mean faecal egg count and both the lower and upper 95% confidence limits. A decreased efficacy was observed in half or more of the farms in Germany, France and the UK. For moxidectin, resistance was confirmed on 3 farms in France, and on 1 farm in Germany and the UK. For ivermectin, resistance was confirmed on 3 farms in the UK, and on 1 farm in Germany and France. The remaining farms with decreased efficacy were classified as having an inconclusive resistance status based on the available data. After treatment *Cooperia* spp. larvae were most frequently identified, though *Ostertagia ostertagi* was also found, in particular within the UK and Germany. The present study reports lower than expected efficacy for ivermectin and moxidectin (based on the reduction in egg excretion after treatment) on European cattle farms, with confirmed anthelmintic resistance on 12.5% of the farms.

## Highlights

• Multi-centre study in four major cattle markets in Europe.
• Decreased efficacy in more than half of the farms in three out of four countries.
• 12.5% of farms with confirmed anthelmintic resistance to ivermectin and moxidectin.
• Mainly *Cooperia* larvae found in post-treatment copro-cultures.
• Also *Ostertagia ostertagi* larvae found in post-treatment copro-cultures.

## Graphical abstract

**Figure:**
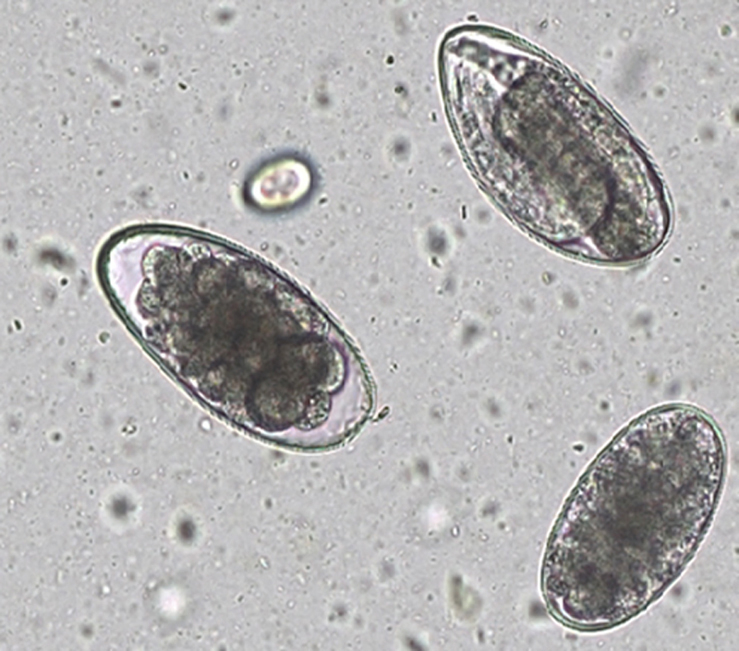

## 1 Introduction

Gastro-intestinal nematodes are very common in cattle and can cause pathology which impairs the health and welfare of infected animals, therefore resulting in decreased production. The use of broad spectrum anthelmintic drugs has been the backbone of worm management for nearly 40 years. This continual use has led to the selection of populations of drug-resistant worms worldwide. The high prevalence of anthelmintic resistance and even multidrug resistance in sheep is redefining how anthelmintics are used in parasite control programs. Anthelmintic resistance to both benzimidazole and macrocyclic lactone compounds has now also been reported in cattle (Sutherland and Leathwick, 2011). For the macrocyclic lactone compounds, anthelmintic resistance in cattle has mainly been reported in the dose-limiting nematode *Cooperia* spp. (Soutello et al., 2007; Condi et al., 2009; Gasbarre et al., 2009; Edmonds et al., 2010; El-Abdellati et al., 2010a,b; McArthur et al., 2011; Bartley et al., 2012; Leathwick and Miller, 2013) and to a far lesser extent in *Ostertagia ostertagi* (Demeler et al., 2009; Areskog et al., 2013). Species belonging to the genus *Cooperia* are considered to be of less pathogenic significance than *Ostertagia*, although the negative effect of *Cooperiapunctata* on cattle productivity has been demonstrated in the US (Stromberg et al., 2012).

The ostensibly lower rate of anthelmintic resistance detection in cattle might in part be due to the difficulty in diagnosis, as faecal egg counts tend to be less reflective of the adult worm burden in cattle compared to sheep. As such, the detection of anthelmintic resistance based on faecal egg counts is prone to more uncertainty in cattle. Some recommendations to minimise diagnostic uncertainty include the use of arithmetic means over geometric means to calculate anthelmintic efficacy (Dobson et al., 2009), the use of individual based group means with pre- and post-treatment individual faecal egg counts (Calvete and Uriarte, 2013), and the preferred use of diagnostic methods with higher analytic sensitivity to minimise inaccuracies in populations with low baseline faecal egg count (Levecke et al., 2011). It is also important to obtain an adequate sample size, although the number of animals with an adequate infection for enrolment can be limited under field conditions. To account for the problems associated with smaller sample sizes, the use of parametric methods such as Markov chain Monte Carlo within a Bayesian framework have been advocated in equine studies for all group sizes smaller than 50 animals, in order to avoid erroneous inference about the true efficacy of anthelmintics in the field (Denwood et al., 2010). Bayesian methods have also more recently been advocated by other authors (Dobson et al., 2012; Torgerson et al., 2014). Non-parametric bootstrapping has also been considered as a potential analysis method (Vidyashankar et al., 2007), but it has been found to be inappropriate for smaller sample sizes (Denwood et al., 2010), and does not produce correct results with observed reductions of 100% (Denwood et al., 2010; Dobson et al., 2012; Torgerson et al., 2014).

In addition to the method of statistical analysis, the interpretation of the efficacy results and the definition of anthelmintic resistance have been debated. The current WAAVP (World Association for the Advancement of Veterinary Parasitology) guidelines (Coles et al., 1992) state that anthelmintic resistance is present when the efficacy is below 95% and when the lower 95% confidence limit is below 90%, and that anthelmintic resistance is suspected if only one of the two criteria applies. More recently, there have been recommendations to include the upper 95% confidence limit in the assessment of the true resistance status (Lyndal-Murphy et al., 2014), which distinguishes between the two situations where anthelmintic resistance is possible but not certain, and where anthelmintic resistance has been confirmed.

The objective of the current study was to obtain more information on anthelmintic resistance in cattle in Europe. The efficacy of injectable formulations of ivermectin and moxidectin was evaluated in naturally infected cattle in four major cattle markets across Europe.

## 2 Materials and methods

### 2.1 Study design

This study was designed as a multi-site field efficacy study in Germany, France, the UK and Italy. The anthelmintic efficacy was assessed in cattle naturally infected with gastro-intestinal nematodes. The study farms were selected based on the previous use of macrocyclic lactones as the backbone for parasite management. On each farm, all animals grazed the same pasture before and throughout the 14 day evaluation period. The study used a randomised complete block design for each farm, with the individual animal as the experimental unit. In accordance with current guidelines, a faecal egg count was used to select animals for treatment. In order to ensure an adequate number of animals with a positive faecal egg count for randomisation (a target of 20 animals), individual faecal samples from 20 to 50 animals were screened at each farm prior to treatment.

On each farm, animals were paired into blocks by descending pre-treatment faecal egg count. Within each block of two, the animals were randomly allocated to one of the two treatment groups. On Day 0, the weight of the allocated animals was determined using a girth tape. Animals were treated with injectable formulations of either ivermectin (Ivomec^®^ Merial) or moxidectin (Cydectin 1%^®^ Zoetis), both at 0.2 mg/kg bodyweight. On Day 14 (±2 days), individual faecal egg counts were performed for all animals selected for treatment. Faecal egg counts were performed using a modified McMaster technique, with a sensitivity of 12.5 eggs per gram of faeces in all countries except France (detection limit: 15 eggs per gram of faeces). Coprocultures were performed for larval identification (Van Wyk and Mayhew, 2013), based on a single bulk sample (before treatment) or a bulk sample per treatment group (after treatment). Laboratory personnel involved in the faecal egg count and/or the larval identification were blinded to the allocation of animals to treatment. The *in vivo* procedures occurred after ethical review and according to state, national, or international regulations.

### 2.2 Statistical analysis

At each farm, the pre-treatment and post-treatment arithmetic mean faecal egg counts were used to calculate the efficacy for each of the treatment groups using the following formula:

$$\text{\%}\;\text{reduction}=100\times \frac{\left(\text{pre}-\text{treatment}\;\text{count}\right)-\left(\text{post}-\text{treatment}\;\text{count}\right)}{\text{pre}-\text{treatment}\;\text{count}}$$

Where possible, the percentage reduction per nematode species (*Ostertagia* and *Cooperia*) was calculated. A bootstrap analysis approach based on Vidyashankar et al. (2007) was used to estimate the arithmetic mean faecal egg count reduction per treatment and the 95% confidence intervals. For each dataset, new values for the pre- and post-treatment counts were sampled with replacement from the observed counts, and the bootstrapped efficacy was calculated as described above. This was repeated 1000 times and the 95% confidence intervals taken as the 2.5 and 97.5 percentiles of the resulting distribution of bootstrapped efficacies. All statistical analyses were performed using R (R Core Team, 2015).

### 2.3 Interpretation of results

The anthelmintic resistance status was interpreted using the method described by Lyndal-Murphy et al. (2014), which is based on the WAAVP guidelines on anthelmintic resistance (Coles et al., 1992) but also considers the upper 95% confidence limit as well as the lower 95% confidence limit and the percentage reduction. The treatment was classified as either efficacious, having confirmed anthelmintic resistance, or being inconclusive based on the following criteria:

• Efficacious: percentage reduction and upper 95% confidence limit above 95% and lower 95% confidence limit above 90%.
• Confirmed anthelmintic resistance: percentage reduction and upper 95% confidence limit below 95% and lower 95% confidence limit below 90%.
• Inconclusive: neither of the above criteria fulfilled.

Datasets with 100% observed efficacy were tentatively classified as efficacious in accordance with these criteria, however these classifications should not be considered as definitive because the lower 95% confidence intervals produced using non-parametric bootstrapping for these datasets cannot be relied upon (Denwood et al., 2010; Dobson et al., 2012; Torgerson et al., 2014).

### 2.4 Comparison of the sampling assumptions and analytical methods

In order to explore the statistical consequences and implications of the standard protocol followed for this study, data from the 80 trials were re-analysed under three sampling assumptions including the *screened* data as described above. To generate the second group of datasets, the entire pre-treatment data were considered. This included data from animals that were not enrolled in the treatment groups due to a low or negative faecal egg count from this sample (*unscreened* data). The motivation was to correct for regression to the mean, induced by using the same faecal egg count sample as both the screening and pre-treatment sample, which would be expected to introduce a bias in the estimated efficacy calculation. The third procedure involved extending the first procedure to utilise the pre-treatment data from both treatment groups in order to reduce the uncertainty in the estimated efficacy (*grouped* data).

All three datasets from each of the 80 trials were analysed using two statistical methods: a modified non-parametric bootstrap method, and an Markov chain Monte Carlo method. The latter was modified from Denwood et al. (2010) to allow an individual animal efficacy fitted to those animals for which a post-treatment faecal egg count was observed. This approach explicitly controls for several levels of within individual extra-Poisson variance, as described by Denwood et al. (2012). These statistical analyses were done in R (R Core Team, 2015), with Markov chain Monte Carlo results obtained using the bayescount package (Denwood, 2015). Full details of these methods are given in the supplementary data file.

## 3 Results

A total of 40 farms and 753 animals were included in this study: 12 farms in Germany with 235 animals, 10 farms each in Italy (179 animals) and the UK (197 animals) and 8 farms (142 animals) in France. In addition to an overview of the efficacy results by country, the number of animals in each country is provided in Table 1. The number of animals enrolled in each treatment group varied between seven and ten (see Tables 2–5). Of the 80 treatment groups in the study, 54 groups had ten animals, 14 groups had nine animals, 6 groups had eight animals, 5 groups had seven animals and 1 group had six animals included in the analysis. For some animals, a faecal sample could not be collected after treatment.

The bulk coprocultures before and after treatment provide further insight into which nematode species are potentially involved in the decreased efficacy or confirmed anthelmintic resistance. The data are presented per farm in Tables 2–5 In Table 6, an overview of the efficacy against *Cooperia* and *Ostertagia*, based on the reduction in mean faecal egg counts and the larval identification before and after treatment is provided.

### 3.1 Germany

The results in Germany are summarised in Table 2. The study was conducted from August 2011 to September 2012 on 12 dairy farms in Northern Germany in the regions of Lower Saxony (GE01-03, GE05-09, GE11), Schleswig–Holstein (GE04 and GE10) and Brandenburg (GE12).

The baseline faecal egg count in Germany was medium to high, with an arithmetic mean faecal egg count between 60 and 836 eggs per gram of faeces, and an individual faecal egg count ranging from 25 to 5587.5 eggs per gram of faeces. Moxidectin was considered to be efficacious on four farms (GE02; GE05; GE10 and GE12). Anthelmintic resistance was confirmed on 1 farm (GE01) and seven cases were found to be inconclusive. In 5 of these cases, the species-specific efficacy calculation (Table 6) indicated that the efficacy was below 95% for *Cooperia* and *Ostertagia* (GE07, GE11) or for *Cooperia* only (GE03, GE04, GE06). Ivermectin was considered to be efficacious on two farms (GE03 and GE04), of which the latter was associated with an observed reduction of 100%. Confirmed anthelmintic resistance was identified on the same farm as for moxidectin (GE01). Nine cases were found to be inconclusive. In 5 of these cases, the species-specific efficacy calculation (Table 6) indicated that the efficacy was below 95% for *Cooperia* and *Ostertagia* (GE07, GE08, GE12) or for *Cooperia* only (GE11, GE09). Before treatment, *O. ostertagi* was identified in all farms and *Cooperia* spp. in all but one of the farms. Small percentages of *Trichostongylus* spp. were also identified (data not shown). Overall, *Cooperia* spp. was the most prevalent nematode after treatment. Nevertheless, *O. ostertagi* was identified in eight of the ten moxidectin and in nine of the ten ivermectin treatment groups from which larvae could be retrieved after treatment.

### 3.2 Italy

The results in Italy are summarised in Table 3. The study was conducted in beef cattle farms from October to December 2012 in the northern part of Italy (Veneto region: IT01-05) and from December 2011 to February 2012 in the southern part of Italy (Apulia region: IT06-10).

The baseline faecal egg count in Italy was low to medium, with an arithmetic mean faecal egg count between 35 and 131 eggs per gram of faeces, and an individual faecal egg count ranging from 25 to 450 eggs per gram of faeces. Moxidectin was considered to be efficacious on nine farms, all of which were associated with a 100% observed reduction. On one farm (IT05) in northern Italy the results were found to be inconclusive. Ivermectin was found to be efficacious on seven farms (two farms in northern Italy and five in southern Italy), all of which were associated with a 100% observed reduction. The results of the remaining three farms (IT01, IT02 and IT03) were inconclusive.

Before treatment *Haemonchus* spp. was the only worm identified on all farms, with one exception (IT01: which also had *Cooperia* and *Oesophagostomum*; data not shown). The only nematode identified after treatment was *Haemonchus* spp.

### 3.3 United Kingdom

The results in the UK are summarised in Table 4. The study was conducted on five dairy farms (UK06-10) in Northumberland from July to September 2011, and five dairy farms (UK02 and UK11-14) in the region of West Sussex from August 2011 to October 2012.

The baseline faecal egg count in the UK was medium to high, with an arithmetic mean faecal egg count between 95 and 722 eggs per gram of faeces, and an individual faecal egg count ranging from 37.5 to 3075 eggs per gram of faeces. It is interesting to note that on farms UK02 and UK14, all treated animals were excreting eggs by 14 days after ivermectin and moxidectin treatment. Moxidectin was considered to be efficacious on four farms (UK09, UK10, UK11 and UK13), of which one farm (UK09) was associated with a 100% observed reduction. Anthelmintic resistance was confirmed on one farm (UK14) and five cases were found to be inconclusive. In 4 of these cases, the species-specific efficacy calculation (Table 6) indicated that the efficacy was below 95% for *Cooperia* and *Ostertagia* (UK02) or for *Cooperia* only (UK07, UK08, UK12). Ivermectin was considered to be efficacious on three farms (UK07, UK09 and UK10), of which 1 farm (UK09) was associated with a 100% observed reduction. Confirmed anthelmintic resistance was identified on three farms (UK02, UK12 and UK14), and the results on four farms were inconclusive. In 3 of these cases, the species-specific efficacy calculation (Table 6) indicated that the efficacy was below 95% for *Cooperia* and *Ostertagia* (UK11, UK13) or for *Cooperia* only (UK08).

Before treatment, coprocultures were successful for nine out of ten farms and both *Cooperia* spp. and *O. ostertagi* were identified. The most prevalent nematode after treatment was *Cooperia* spp. although *O. ostertagi* was also identified on seven farms after moxidectin treatment (10–94% of the larvae) and on five farms after ivermectin treatment (6–100% of the larvae). On one farm (UK06), 94% of the larvae after moxidectin treatment and 100% of the larvae after ivermectin treatment were identified as *O. ostertagi*.

### 3.4 France

The results of the eight dairy farms in France are summarised in Table 5. The study was conducted from November 2011 to September 2012, in the region of Nantes.

The baseline faecal egg count in France was low to medium, with an arithmetic mean faecal egg count between 40 and 216 eggs per gram of faeces, and an individual faecal egg count ranging from 15 to 1125 eggs per gram of faeces. Moxidectin was considered as efficacious on three farms (FR01, FR05 and FR08), all of which were associated with a 100% observed reduction. Confirmed anthelmintic resistance was observed on three farms (FR06, FR09 and FR12), and on two farms the results were inconclusive. Ivermectin was efficacious on four farms (FR01, FR08, FR09 and FR11), of which three farms (FR01, FR08 and FR11) were associated with a 100% observed reduction. Anthelmintic resistance was confirmed for ivermectin on one farm (FR12), and on three farms the results were inconclusive. The species-specific efficacy calculation (Table 6) indicated that the efficacy was below 95% for *Cooperia* only (FR06).

Before treatment, coprocultures were successful for seven out of eight farms: *Cooperia* spp. was identified on seven farms and *O. ostertagi* was identified on five farms. The most prevalent nematodes after treatment were *Cooperia* spp. In France, only a small percentage of *O. ostertagi* larvae were identified after ivermectin treatment on FR09. The coproculture of FR02 and FR11 did not yield any larvae.

### 3.5 Comparison of the sampling assumptions and analytical methods

There was no significant difference between the observed efficacy from the 80 trials as calculated from the *grouped* and *unscreened* datasets, although the additional pre-treatment data reduced the uncertainty in the true reduction. This was reflected in smaller 95% confidence intervals and therefore fewer datasets classified as inconclusive for the *grouped* data compared to the *unscreened* data (one fewer for Markov chain Monte Carlo and five fewer for the bootstrap method). However, the *screened* datasets significantly over-estimated the efficacy (compared to the *grouped* datasets) by an average of 2.4% over the 80 trials, and for the bootstrap method in particular, were also associated with a tendency to classify trials as efficacious or inconclusive rather than inconclusive or confirmed anthelmintic resistance. Each of the 25 trials with 100% observed reductions were classified as inconclusive by the Markov chain Monte Carlo method using *screened*, *unscreened* and *grouped* datasets, indicating that the non-parametric bootstrap classification of efficacious produced by the non-parametric bootstrap is not reliable for these datasets. Based on the *grouped* data from the remaining 55 trials with observed reductions of less than 100%, 19 were classified as confirmed anthelmintic resistance by both Markov chain Monte Carlo and bootstrap, 26 as inconclusive by both methods, 3 as inconclusive by bootstrap but confirmed anthelmintic resistance by Markov chain Monte Carlo, and 7 as efficacious by bootstrap but inconclusive by Markov chain Monte Carlo. Full results of the comparison between the analytical methods are given in the Supplementary data file.

## 4 Discussion

This study provides an insight into the current state of efficacy of injectable macrocyclic lactone anthelmintics on commercial cattle farms in Germany, Italy, France and the UK. The efficacy in this study was calculated based on arithmetic group means before and after treatment, and was evaluated taking both the lower and upper 95% confidence limit into account. This allowed the definition of three categories: efficacious, confirmed anthelmintic resistance or inconclusive (Lyndal-Murphy et al., 2014). As the criteria are based on upper and lower 95% confidence intervals as well as a calculated percent reduction, the conclusions are dependent on accurate estimates obtained using an appropriate statistical method. In the main text of this paper, standard non-parametric bootstrap methods have been used to estimate the lower and upper 95% confidence intervals. Based on these estimates and on the interpretation as suggested by Lyndal-Murphy et al. (2014), a considerable number of groups were classified as inconclusive. Without consideration of the upper 95% confidence interval these groups would be incorrectly classified as either suspected or confirmed anthelmintic resistance.

Using the Markov chain Monte Carlo method presented in the supplementary file to generate 95% confidence intervals, an even larger number of groups were classified as inconclusive. This illustrates the difficulties in accurately evaluating anthelmintic efficacy based on faecal egg counts in cattle, especially in bovine populations with a low baseline faecal egg excretion. It is also important to note that non-parametric bootstrapping is unable to deal appropriately with an observed reduction of 100% (Denwood et al., 2010; Dobson et al., 2012; Torgerson et al., 2014) and can therefore yield incorrect conclusions in these situations. It is concerning that no groups were classified as efficacious by the Markov chain Monte Carlo method, which suggests that larger sample sizes are required to confidently determine situations where the true efficacy is greater than 95%. Nevertheless, a number of farms with confirmed anthelmintic resistance were consistently identified, as discussed below.

The selection of the farms in this study was based on their previous use of macrocyclic lactone compounds which may have led to an increased selection for the development of anthelmintic resistance. However, the main emphasis of this study was not to estimate the true prevalence of anthelmintic resistance on cattle farms in the respective geographical regions. Instead, this study intended to evaluate the current efficacy situation on farms that were selected to be representative of the relevant market, and therefore regularly use macrocyclic lactones. The threshold for adequate efficacy was set at 95% in this study, in line with the recommendations for macrocyclic lactones (Coles et al., 1992, 2006). Nevertheless, this general threshold does not take potential differences in regional management practices or climate factors into account, and might therefore underestimate the anthelmintic resistance status in regions with high baseline efficacy. In Italy for example (Geurden et al., 2014; Rinaldi et al., 2014), high efficacy of anthelmintics are commonly reported in sheep, in contrast to other EU countries, suggesting a higher threshold for efficacy might be required. It was however preferred to use a common threshold for all countries included, for consistency in this multi-centre study. Specifically for Italy, this general threshold is not believed to have resulted in an underestimation of the resistance status, given the low number of cattle excreting eggs after treatment.

The treatment efficacy in our study was evaluated based on faecal egg count rather than worm counts, because the study animals were owned by the farmers. There are a number of limitations associated with anthelmintic efficacy evaluation based on faecal egg counts, which have been discussed before (Dobson et al., 2009; Denwood et al., 2010; Levecke et al., 2011, 2012). In the present study, a diagnostic method with a low detection limit was used to minimise the problems caused by populations with a low to medium baseline faecal egg count. However, even with this detection limit, identifying animals with an adequate infection proved challenging, reflecting the low baseline faecal egg count commonly observed in cattle in Europe. As a result, up to 50 animals had to be screened on each farm prior to treatment in order to reach the target number of 10 animals in the majority of the treatment groups (88% of the treatment groups had 9 or 10 animals). The sample group sizes are in line with previous studies in Europe (Demeler et al., 2009; Areskog et al., 2013), and with the WAAVP guidance on anthelmintic resistance evaluation (Coles et al., 1992). However, larger group sizes are known to increase the accuracy of the efficacy evaluation, which has been noted by several authors using more sophisticated methods of analysis than those recommended in the current WAAVP guidelines (Gill et al., 1986; Denwood et al., 2010; Levecke et al., 2012).

Moxidectin was found to be efficacious on 20 and ivermectin on 16 out of 40 farms (including the datasets with 100% reduction). In all countries except Italy, a high number of farms with decreased efficacy were observed (ivermectin; n = 24 and moxidectin; n = 20). Some of these cases with decreased efficacy were listed as inconclusive, as it is not possible to classify them as fully effective or as confirmed anthelmintic resistance based on the available faecal egg count data. Looking however into the efficacy calculation for each parasite, it is clear that at least part of those inconclusive cases concern a species-specific efficacy below the efficacy threshold for macrocyclic lactones. The adequate or near to adequate efficacy against one nematode species sometimes masks the clear resistance against the other species, and might lead to an underestimation of species-specific resistance cases. Despite this observation, confirmed anthelmintic resistance was found in 5 farms (12.5%) each for moxidectin and ivermectin. In total, 7 farms (17.5%) had confirmed anthelmintic resistance in at least one of the treatment groups, and on 3 farms (7.5%) confirmed anthelmintic resistance was identified in both groups (GE01, UK14 and FR12), confirming previous observations that ivermectin and moxidectin share some degree of cross-resistance, but not complete cross-resistance (Prichard et al., 2012). It is currently thought that both drugs act via irreversibly opening of the GABA and glutamate gate-chloride channels leading to a flaccid paralysis. However, it is unclear which resistance mechanisms apply and it has been discussed whether drug-specific differences in the involved mechanisms (e.g concerning transmembrane P-glycoproteins), may contribute to different drug efficacy phenotypes (Bygarski et al., 2014).

In Italy, decreased efficacy for ivermectin (associated with the nematode *Haemonchus* spp.) was identified on three farms, although the results were inconclusive. In cattle, anthelmintic resistance against *Haemonchus* has previously been reported in the US (Gasbarre et al., 2009) and in Brazil (Soutello et al., 2007).

Anthelmintic resistance was confirmed in France for both ivermectin and moxidectin on 1 and 3 farms respectively, but this was mainly associated with the dose-limiting nematode species *Cooperia* spp. In contrast, anthelmintic resistance in the UK and Germany was due not only to the dose-limiting nematode species, but also to the more pathogenic abomasal nematode *O. ostertagi,* which was also identified in cases of confirmed anthelmintic resistance. Calculating the species-specific efficacy, confirms that reduced efficacy is mainly observed in *Cooperia*, although in 25% of the farms for which this calculation could be done, the efficacy of both ivermectin and moxidectin against *Ostertagia* was below 95% (all in the UK and Germany). This is in contrast to previous efficacy evaluations of macrocyclic lactone compounds under European field conditions, in which decreased efficacy was mostly associated with *Cooperia* (Demeler et al., 2009; Bartley et al., 2012). In a recent study in Sweden, *O. ostertagi* was identified on 15% of farms after ivermectin or doramectin treatment with topical formulations (Areskog et al., 2013).

The suppression of the egg excretion by *Cooperia* spp. worms following moxidectin treatment has been described (Condi et al., 2009; de Graef et al., 2012; Lopes et al., 2014), potentially leading to an overestimation of the moxidectin efficacy against *Cooperia* spp. based on faecal examination. However, where *Cooperia* spp. larvae were identified after treatment with ivermectin on our study farms, they were also observed on the same farm (and in similar proportions) after treatment with moxidectin. The comparable proportions after treatment seem to suggest that on farms with decreased efficacy of ivermectin against *Cooperia*, the efficacy of moxidectin was not overestimated due to a suppressed egg excretion.

The results of the current study indicate that anthelmintic resistance is an increasing reality in three major cattle rearing countries in Europe, and that this concerns not only the dose-limiting species but also *O. ostertagi*. This should incite the different stakeholders, farmers as well as vets and industry, to work towards what has been referred to as a paradigm shift in parasite control (McArthur and Reinemeyer, 2014). The key message is that management advice should be based on a sound understanding of the parasite epidemiology and farm management, past and present, and that the requirement for and appropriate timing of treatment should be established based on faecal examinations. In the present study, screening of up to 50 animals was needed to identify a sufficient number of animals with an adequate infection prior to treatment. This indicates that not all animals need treatment at the same time and that an approach with targeted treatments could be considered. Different approaches, including Targeted Selective Treatments programs, to decrease the selection pressure for the development of anthelmintic resistance in ruminants, have previously been discussed (Knox et al., 2012; Charlier et al., 2014). The use of combination anthelmintics has also been advocated, in order to increase the farm treatment efficacy and consequently prevent the introduction of resistance genes into the worm population (Smith, 2014) and also to protect any new anthelmintic compound (Bartram et al., 2011; Geary et al., 2012; Knox et al., 2012). Nevertheless, any new combination anthelmintic must be used in a sustainable manner (Lanusse et al., 2014).

## Conflicts of interest

At the time of the study Thomas Geurden, Hima Bindu Vanimisetti and David J. Bartram were paid employees of Zoetis, the study Sponsor.

Thomas Geurden, Hima Bindu Vanimisetti and David J. Bartram have been involved in the study design and implementation, as well as in the data analysis and reporting. They have not been involved in the data collection and examination.

## Figures and Tables

**Table 1:** The number of farms with adequate efficacy (EFF), confirmed anthelmintic resistance (CAR) or inconclusive (INC) for ivermectin (IVM) and moxidectin (MOX), and the number of farms with CAR for both IVM and MOX. The number of farms (N farms), and the number of animals per treatment group (N animals) are provided per country.

Country	N farms	MOX				IVM				CAR for IVM and MOX
N animals	EFF	CAR	INC	N animals	EFF	CAR	INC
Germany	12	118	4	1	7	117	2	1	9	2
Italy	10	88	9	0	1	91	7	0	3	0
UK	10	99	4	1	5	98	3	3	4	4
France	8	71	3	3	2	71	4	1	3	4
Total	40	376	20	5	15	377	16	5	19	10

**Table 2:** The arithmetic mean (AM), minimum (MIN) and maximum (MAX) faecal egg counts before (pre) and after (post) treatment for each treatment group on the 12 farms (GE01-12) in Germany. The number of animals in each treatment group (n) and the number of animals with a positive faecal egg count after treatment (n pos) are provided, along with the % larvae before and after treatment (Oo = *Ostertagia ostertagi* and C = *Cooperia* spp). The percentage (%) efficacy is provided calculated based on the arithmetic group mean, along with the lower (L95) and upper (U95) 95% confidence limits. The anthelmintic resistance status (Status) is provided as EFF (efficacious), confirmed anthelmintic resistance (CAR) or Inconclusive (INC).

Farm	Treatment	Pre				Post				Efficacy				Larvae pre		Larvae post	
n	AM	MIN	MAX	n pos	AM	MIN	MAX	%	L95	U95	Status	Oo	C	Oo	C
GE01	MOX	10	260	75	550	8	58	0	187.5	76.8	56.9	90.4	CAR	44	56	3	97
IVM	10	258	62.5	475	9	55	0	150	78.3	63.3	88.4	CAR	3	97
GE02	MOX	10	75	25	187.5	2	3	0	12.5	96.5	91.3	100.0	EFF	100	0	100	0
IVM	10	75	37.5	175	2	4	0	25	94.9	86.8	100.0	INC	90	10
GE03	MOX	10	133	12.5	625	3	9	0	62.5	92.0	75.0	100.0	INC	77	22	0	100
IVM	10	109	12.5	475	1	1	0	12.5	98.7	94.6	100.0	EFF	NL	
GE04	MOX	10	89	25	325	1	4	0	37.5	95.5	82.4	100.0	INC	40	60	0	100
IVM	10	129	25	587.5	0	0	0	0	100.0	100.0	100.0	EFF	0	100
GE05	MOX	10	836	62.5	5587.5	6	15	0	37.5	97.3	91.1	99.5	EFF	20	80	2	95
IVM	10	465	37.5	2212.5	5	18	0	75	95.2	85.7	99.1	INC	2	98
GE06	MOX	10	106	12.5	387.5	5	23	0	125	77.2	37.3	96.7	INC	21	79	5	95
IVM	10	96	12.5	262.5	2	4	0	25	95.7	87.3	100.0	INC	5	95
GE07	MOX	8	78	12.5	225	4	8	0	25	89.0	76.5	97.4	INC	4	95	6	94
IVM	7	75	25	200	3	7	0	25	89.9	73.9	98.4	INC	30	67
GE08	MOX	10	69	25	150	3	4	0	12.5	94.4	88.0	98.6	INC	91	6	NL	
IVM	10	66	12.5	137.5	2	20	0	175	69.6	8.3	100.0	INC	24	76
GE09	MOX	10	64	12.5	212.5	4	9	0	25	85.0	64.7	97.3	INC	43	57	NL	
IVM	10	60	12.5	162.5	2	3	0	12.5	95.7	88.6	100.0	INC	10	90
GE10	MOX	10	101	37.5	237.5	2	3	0	12.5	97.5	93.8	100.0	EFF	31	69	13	87
IVM	10	74	12.5	175	3	5	0	25	92.8	83.3	98.6	INC	NL	
GE11	MOX	10	103	50	175	4	13	0	50	87.5	75.0	97.1	INC	44	56	29	71
IVM	10	114	50	250	3	5	0	25	95.5	89.6	100.0	INC	9	91
GE12	MOX	10	86	37.5	200	3	4	0	12.5	95.4	90.0	99.2	EFF	35	64	13	88
IVM	10	129	37.5	650	5	66	0	450	36.6	0.00	95.3	INC	20	80

NL = no larvae were found in the coproculture. MOX = moxidectin injectable. IVM = ivermectin injectable.

**Table 3:** The arithmetic mean (AM), minimum (MIN) and maximum (MAX) faecal egg counts before (pre) and after (post) treatment for each treatment group on the 10 farms (IT01-10) in Italy. The number of animals in each treatment group (n) and the number of animals with a positive faecal egg count after treatment (n pos) are provided, along with the % larvae before and after treatment (Hae = *Haemonchus* spp. and C = *Cooperia* spp). The percentage (%) efficacy calculated based on the arithmetic group mean is provided, along with the lower (L95) and upper (U95) 95% confidence limits. The anthelmintic resistance status (Status) is provided as EFF (efficacious), confirmed anthelmintic resistance (CAR) or Inconclusive (INC).

Farm	Treatment	Pre				Post				Efficacy				Larvae pre		Larvae post	
n	AM	MIN	MAX	n pos	AM	MIN	MAX	%	L95	U95	Status	Hae	C	Hae	C
IT01	MOX	10	63	25	125	0	0	0	0	100	100.0	100.0	EFF	78^a^	18^a^		
IVM	9	97	50	400	3	19	0	100	77.1	36.3	100.0	INC	100	0
IT02	MOX	9	111	25	400	0	0	0	0	100	100.0	100.0	EFF	100	0		
IVM	9	131	25	450	2	6	0	25	95.4	87.1	100.0	INC	100	0
IT03	MOX	6	42	25	75	0	0	0	0	100	100.0	100.0	EFF	100	0		
IVM	8	41	25	100	1	6	0	50	84.6	50.0	100.0	INC	100	0
IT04	MOX	6	42	25	75	0	0	0	0	100	100.0	100.0	EFF	100	0	ND	
IVM	8	50	25	200	0	0	0	0	100	100.0	100.0	EFF
IT05	MOX	7	64	25	225	1	4	0	25	93.4	71.4	100.0	INC	100	0	NL	
IVM	7	39	25	100	0	0	0	0	100	100.0	100.0	EFF		
IT06	MOX	10	38	25	62.5	0	0	0	0	100	100.0	100.0	EFF	100	0	ND	
IVM	10	40	25	112.5	0	0	0	0	100	100.0	100.0	EFF
IT07	MOX	10	49	25	125	0	0	0	0	100	100.0	100.0	EFF	100	0	ND	
IVM	10	46	25	100	0	0	0	0	100	100.0	100.0	EFF
IT08	MOX	10	46	25	87.5	0	0	0	0	100	100.0	100.0	EFF	100	0	ND	
IVM	10	48	25	87.5	0	0	0	0	100	100.0	100.0	EFF
IT09	MOX	10	38	25	75	0	0	0	0	100	100.0	100.0	EFF	100	0	ND	
IVM	10	35	25	50	0	0	0	0	100	100.0	100.0	EFF
IT10	MOX	10	46	25	137.5	0	0	0	0	100	100.0	100.0	EFF	100	0	ND	
IVM	10	45	25	112.5	0	0	0	0	100	100.0	100.0	EFF

ND = for those groups with high efficacy no copro-culture was performed. NL = no larvae were found in the coproculture.

MOX = moxidectin injectable. IVM = ivermectin injectable.

a On IT01, *Oesophagostomum* (4%) was identified as well pre-treatment.

**Table 4:** The arithmetic mean (AM), minimum (MIN) and maximum (MAX) faecal egg counts before (pre) and after (post) treatment for each treatment group on the 10 farms (UK02 and UK06-14) in the UK. The number of animals in each treatment group (n) and the number of animals with a positive faecal egg count after treatment (n pos) are provided, along with the % larvae before and after treatment (Oo = *Ostertagia ostertagi* and C = *Cooperia* spp). The percentage (%) efficacy calculated based on the arithmetic group mean is provided, along with the lower (L95) and upper (U95) 95% confidence limits. The anthelmintic resistance status (Status) is provided as EFF (efficacious), confirmed anthelmintic resistance (CAR) or Inconclusive (INC).

Farm	Treatment	Pre				Post				Efficacy				Larvae pre		Larvae post	
n	AM	MIN	MAX	n pos	AM	MIN	MAX	%	L95	U95	Status	Oo	C	Oo	C
UK02	MOX	10	353	37.5	800	10	35	12.5	125	89.8	80.8	95.3	INC	10	47	17	68
IVM	10	320	12.5	862.5	10	61	12.5	237.5	79.3	55.6	92.5	CAR	0	99
UK06	MOX	10	243	162.5	412.5	6	21	0	62.5	91.2	84.5	96.9	INC	NL		94	6
IVM	10	234	150	350	6	13	0	50	94.6	90.6	98.0	INC	100	0
UK07	MOX	10	254	87.5	762.5	2	9	0	75	96.4	89.6	100.0	INC	41	52	0	100
IVM	10	223	75	400	2	3	0	12.5	98.8	97.0	100.0	EFF	0	90
UK08	MOX	10	170	62.5	625	7	14	0	25	91.2	83.9	96.0	INC	57	40	16	84
IVM	10	126	62.5	337.5	5	29	0	187.5	76.8	41.3	96.3	INC	6	94
UK09	MOX	10	95	50	175	0	0	0	0	100.0	100.0	100.0	EFF	69	17	NL	
IVM	10	105	50	250	0	0	0	0	100.0	100.0	100.0	EFF
UK10	MOX	10	216	75	437.5	2	3	0	12.5	98.8	97.1	100.0	EFF	21	55	0	100
IVM	10	303	137.5	937.5	1	1	0	12.5	99.6	98.6	100.0	EFF	0	100
UK11	MOX	9	722	212.5	3075	4	18	0	50	97.1	92.1	99.5	EFF	21	78	32	68
IVM	9	546	187.5	2162.5	6	63	0	175	86.8	67.4	97.2	INC	29	70
UK12	MOX	10	391	125	925	6	21	0	137.5	94.5	86.2	98.8	INC	56	44	10	90
IVM	10	388	125	687.5	7	56	0	175	85.0	72.7	95.0	CAR	18	82
UK13	MOX	10	299	150	737.5	5	11	0	62.5	96.2	91.3	99.1	EFF	26	74	21	79
IVM	9	351	137.5	1137.5	5	63	0	450	80.7	44.4	98.4	INC	38	61
UK14	MOX	10	149	62.5	587.5	10	18	12.5	50	87.1	75.3	94.1	CAR	40	60	27	67
IVM	10	113	50	262.5	10	60	12.5	287.5	46.4	−12.5	83.3	CAR	0	100

NL = no larvae were found in the coproculture. MOX = moxidectin injectable. IVM = ivermectin injectable.

**Table 5:** The arithmetic mean (AM), minimum (MIN) and maximum (MAX) faecal egg counts before (pre) and after (post) treatment for each treatment group on the 8 farms (FR01-12) in France. The number of animals in each treatment group (n) and the number of animals with a positive faecal egg count after treatment (n pos) are provided, along with the % larvae before and after treatment (Oo = *Ostertagia ostertagi* and C = *Cooperia* spp). The percentage (%) efficacy calculated based on the arithmetic group mean is provided, along with the lower (L95) and upper (U95) 95% confidence limits. The anthelmintic resistance status (Status) is provided as EFF (efficacious), confirmed anthelmintic resistance (CAR) or Inconclusive (INC).

Farm	Treatment	Pre				Post				Bootstrap				Larvae pre		Larvae post	
n	AM	MIN	MAX	n pos	AM	MIN	MAX	%	L95	U95	Status	Oo	C	Oo	C
FR01	MOX	9	40	15	90	0	0	0	0	100	100.0	100.0	EFF	91	9	ND	
IVM	9	57	15	165	0	0	0	0	100	100.0	100.0	EFF	ND	
FR04	MOX	7	41	15	75	2	9	0	45	77.5	37.5	100.0	INC	76	24	NL	
IVM	9	38	15	75	4	12	0	45	68.3	35.0	95.0	INC
FR05	MOX	8	168	15	465	0	0	0	0	100	100.0	100.0	EFF	0	100	ND	
IVM	7	216	15	735	2	9	0	45	95.2	82.5	100.0	INC	ND	
FR06	MOX	9	110	15	315	6	47	0	135	52.6	−12.1	86.1	CAR	6	94	0	100
IVM	9	138	15	600	3	37	0	210	68.5	2.5	98.1	INC	0	100
FR08	MOX	9	137	30	420	0	0	0	0	100	100.0	100.0	EFF	12	88	ND	
IVM	9	170	30	465	0	0	0	0	100	100.0	100.0	EFF	ND	
FR09	MOX	10	74	15	165	6	21	0	45	70.1	43.9	87.7	CAR	NL		0	100
IVM	10	176	15	1125	1	2	0	15	98.8	94.3	100.0	EFF	6	94
FR11	MOX	9	75	15	210	2	5	0	30	92.7	78.9	100.0	INC	5	95	NL	
IVM	8	84	15	240	0	0	0	0	100	100.0	100.0	EFF
FR12	MOX	10	167	60	345	8	50	0	195	68.8	39.6	88.7	CAR	0	100	0	100
IVM	10	161	75	345	5	36	0	120	77.3	57.6	94.1	CAR	0	100

ND = for those groups with high efficacy no copro-culture was performed. NL = no larvae were found in coproculture. MOX = moxidectin injectable. IVM = ivermectin injectable.

**Table 6:** The percentage efficacy calculated for *Cooperia* and *Ostertagia* separately for those treatment groups on those farms where larval identification was available before and after treatment (IVM = ivermectin; MOX = moxidectin). The number of groups with efficacy lower than 95% is provided (<95%).

	*Cooperia* spp		*Ostertagia* spp	
Farm	IVM	MOX	IVM	MOX
GE01	63%	61%	99%	98%
GE02			95%	96%
GE03		69%		100%
GE04	100%	93%	100%	100%
GE05	95%	98%	100%	100%
GE06	95%	74%	99%	95%
GE07	93%	90%	30%	85%
GE08	−284%		92%	
GE09	92%		99%	
GE10		96%		99%
GE11	93%	84%	99%	92%
GE12	36%	94%	71%	98%
UK02	60%	86%	100%	83%
UK07	98%	93%	100%	100%
UK08	46%	83%	98%	98%
UK10	99%	97%	100%	100%
UK11	90%	98%	84%	96%
UK12	73%	89%	95%	99%
UK13	85%	96%	74%	97%
UK14	12%	87%	100%	92%
FR06	71%	55%	100%	100%
FR12	66%	70%	100%	100%
<95%	14/19	13/19	5/20	5/20
