# Supplementary material for: Anthelmintic resistance to ivermectin and moxidectin in gastrointestinal nematodes of cattle in Europe
Source: Int J Parasitol Drugs Drug Resist. 2015 Aug 18;5(3):163–71. doi: 10.1016/j.ijpddr.2015.08.001 (PMC4572401; doi:10.1016/j.ijpddr.2015.08.001)
Supplement: Supplementary file 1 [file mmc1.docx]

**Supplementary file to ‘Anthelmintic resistance to ivermectin and moxidectin in gastrointestinal nematodes of cattle in Europe’: Comparison of the sampling assumptions and analytical methods**

**Data Handling**

The data collection outlined in the main paper involved a screening faecal egg count applied to all animals within a treatment block to determine which individuals are included in the efficacy evaluation. In the study, the faecal egg count from the screening sample was used as the pre-treatment sample for the enrolled animals, and the screening faecal egg counts from those animals that were not enrolled were not retained for analysis. The dataset with only the faecal egg counts for enrolled animals is referred to as the *screened* dataset.

An alternative approach is to include all observed pre-treatment screening data, with the missing corresponding post-treatment data handled using an appropriate statistical method. These data including all observed pre-treatment screening data are referred to as the *unscreened* dataset. Note that for datasets where all available animals were allocated to a treatment group, the *unscreened* data is identical to the *screened* data.

For this study, two anthelmintics were tested on each farm, with individual animals randomly allocated to a treatment group. As a result, the pre-treatment data from both treatment groups can be considered as a single distribution of pre-treatment faecal egg counts, and can therefore be included in the calculation of efficacy for each treatment. This approximately doubles the sample size for the pre-treatment data. We refer to this data as the *grouped* data.

To clarify the difference between these datasets, consider the example given in Table S1.

**Statistical Methods**

The statistical methods used to analyse the *screened* datasets are as described in the main text. In order to allow for missing post-treatment faecal egg counts in the *unscreened* data, the procedures were modified as follows.

For the Markov chain Monte Carlo method, the latent pre-treatment mean of each animal was modelled as a gamma distribution around the group mean, with the observed pre-treatment faecal egg counts modelled as a compound gamma-Poisson distribution conditional on the unobserved pre-treatment mean egg counts for these animals. The available post-treatment faecal egg counts were modelled as a second gamma-Poisson distribution conditional on the corresponding pre-treatment animal mean and the estimated group reduction, with the missing post-treatment faecal egg count data imputed as part of the same distribution. This model is less computationally efficient than the method described by Denwood *et al*. (2010), but allows for non-random missing post-treatment data by explicitly modeling the faecal egg count reduction as a process within individual animals, allowing for within-individual extra-Poisson variance as described by Denwood *et al*. (2012). To ensure that the underlying assumptions of the count distribution were met, all data were transformed from eggs per gram of faeces to the raw counts (number of eggs observed) before analysis using the Markov chain Monte Carlo methods.

For the bootstrap method, missing faecal egg counts in the post-treatment data were imputed based on the corresponding pre-treatment faecal egg counts and a randomly sampled observed reduction from another individual within the same treatment group. This sampling was updated at each iteration and the confidence intervals obtained from the 1,000 bootstrapped reductions as described in the main text. For the *grouped* dataset, the post-treatment faecal egg counts for individuals not assigned to the treatment group being analysed were regarded as missing, and the same procedure followed for both the Markov chain Monte Carlo and bootstrap methods as outlined above.

The data from the 80 trials described in the main text were processed into 80 sets of three corresponding *screened*, *unscreened* and *grouped* datasets, and analysed using bootstrap and Markov chain Monte Carlo methods. All statistical analyses for the supplementary file were implemented in R (R Core Team, 2015), with Markov chain Monte Carlo results obtained using the bayescount package (Denwood, 2015). The interpretation of all confidence intervals was as described in the main text, and R code for all analyses run in the appendix is available upon request from the authors.

**Results**

There was an average difference in efficacy of 0.1% (range -18.4% to 9.3%) calculated from the 80 unscreened datasets compared to the 80 grouped datasets. This small difference was not found to be significant using a paired Wilcoxon test (p=0.23). In contrast, the efficacy calculated using the *screened* datasets differed to the grouped datasets by an average of ‑2.4% (range ‑36.8% to 10.3%), and was statistically significant (p<0.001). This demonstrates that the *screened* datasets tend to result in an overestimation of the drug efficacy, relative to the other data-handling methods.

The 25 trials with 100% observed reductions were all classified as inconclusive by the Markov chain Monte Carlo method using *screened*, *unscreened* and *grouped* datasets. For the remaining 55 trials with observed reductions of less than 100%, there was perfect agreement between analyses from each of the three corresponding datasets for 49 trials (CAR=17; INC=32; EFF=0) when analysed using Markov chain Monte Carlo, but only 39 trials (CAR=11; INC=23; EFF=5) had the same conclusion when each was analysed using the bootstrap method. Agreement between corresponding *grouped* and *unscreened* datasets was almost perfect for Markov chain Monte Carlo, except for a single dataset, which was classified as inconclusive with the unscreened dataset but confirmed anthelmintic resistance with the grouped dataset. A total of 9 trials showed disagreement between *grouped* and *unscreened* datasets for the bootstrap method. All 27 trials with conflicting conclusions from at least one method/dataset combination are shown in Table S2.

When comparing the *screened* datasets to the *grouped* datasets, a single *screened* dataset classified as efficacious by Markov chain Monte Carlo was reclassified as inconclusive, and 5 *screened* datasets classified as inconclusive were reclassified as confirmed resistant. This consistent shift towards lower confidence in the efficacy of the drug is in line with the findings regarding bias for the *screened* datasets as discussed above. However, this pattern was not as consistent with the bootstrap method. Of the 33 *screened* datasets classified as inconclusive by bootstrap, 8 were reclassified as confirmed anthelmintic resistance, yet 2 were reclassified as efficacious, and 6 of the 11 datasets classified as efficacious by bootstrap were reclassified as inconclusive based on the *grouped* data. As a result, the disagreement between Markov chain Monte Carlo and bootstrap methods was greater for the *screened* data (20 of the 55 farms) than for the *grouped* data (10 of the 55 farms).

**Discussion**

As demonstrated in this supplementary file, the statistical method chosen to analyse faecal egg count reduction data can affect the confidence intervals produced, and therefore the conclusions drawn from the data. However, we have also demonstrated that the way in which the data are handled before analysis can have a more profound effect on the conclusions. Using screening faecal egg counts to select animals for treatment is a standard procedure, motivated by the desire to reduce the rate of development of anthelmintic resistance by reserving anthelmintic dosing for those animals shown to be shedding parasite eggs. This practice also helps to avoid an excessive number of pre-treatment samples with zero counts, which present difficulties for some non-parametric statistical analysis methods. However, we have demonstrated that reusing these same samples as the pre-treatment faecal egg counts results in a bias towards higher calculated efficacy, particularly when using the bootstrap method. This occurs because some of the variability in observed faecal egg counts arises due to within-animal sources, such as technical variability due to the process of counting eggs and variability in egg shedding within an individual animal. By applying a threshold for selection to these data, a bias is introduced towards the selection of higher pre-treatment faecal egg counts from individual animals that may, in fact, have the same long-run mean faecal egg counts as individuals who were not selected for treatment on the basis of a single observation of low faecal egg count. The unbiased post-treatment samples will therefore be lower on average than their biased corresponding pre-treatment samples because of regression to the mean, resulting in an over-estimate of the calculated drug efficacy. We therefore advocate the use of a statistical analysis method capable of handling missing post-treatment data, such as those presented here.

By using all available pre-treatment data, the methods demonstrated here have the further advantage of reducing the uncertainty in the estimated efficacy, bringing the upper and lower 95% confidence intervals closer to the mean, and therefore reducing the probability of an inconclusive result. However, even when using the *grouped* data, the Markov chain Monte Carlo method determined that the results were inconclusive for 58 of the 80 trials (72.5%), and the bootstrap method determined that the results were inconclusive for 29 of the 55 trials (53%) where the observed reduction was less than 100%. This indicates that the current recommendations regarding sample sizes are not sufficient to reliably produce conclusive results. Larger group sizes are known to increase the accuracy in efficacy evaluation, but are not always feasible due to limitations in the number of animals available for treatment. However, relatively complex statistical methods such as Markov chain Monte Carlo are able to incorporate multiple faecal egg counts from the same animal (Denwood *et al*., 2012), and therefore have the potential to increase the study power. Some possible methods of performing power calculations for faecal egg count reduction tests were first published almost 30 years ago (Gill *et al*., 1986), but they have not been widely applied. Further work in this area is therefore required.

Neither of the statistical methods presented here are without criticism. Parametric bootstrapping has the advantage of relative simplicity, both conceptually and in terms of application, and does not make any distributional assumptions about the data. However, this method assumes that the data observed is fully representative of all possible observed data, and has been shown to be unreliable with sample sizes of less than approximately 50 animals (Denwood *et al*., 2010). This is most clearly demonstrated for datasets with a 100% observed reduction, for which non-parametric bootstrapping methods cannot produce appropriate confidence intervals (Denwood *et al*., 2010; Dobson *et al.,* 2012; Torgerson *et al.,* 2014). Conversely, parametric methods such as Markov chain Monte Carlo are useful for datasets with small sample sizes or 100% observed reductions, and also appear to be more robust to the biasing effects of the *screened* data than the bootstrap method for the data presented here. However, the relative complexity of Markov chain Monte Carlo may be a barrier to its implementation within parasitology, although the technique has been widely adopted in other fields. The requirement for Bayesian methods to use prior information is also a potential area of criticism, and tends to draw the mean estimate produced by the Markov chain Monte Carlo method towards the centre of the prior (corresponding to an efficacy of 50% in this case). For datasets typical of parasitology, this will tend to reduce the mean efficacy estimate. However, the effect of this ‘minimally informative’ prior is not strong, and different prior distributions can be substituted where appropriate. Given the inherent difficulties associated with analysing typical faecal egg count reduction data, we advocate the more complex yet more robust parametric methods over the simpler, non-parametric bootstrap method. The modified Markov chain Monte Carlo and bootstrap methods required to analyse these data are available within the bayescount R package (Denwood, 2015), and interested readers are encouraged to contact the author of the package for more information.

**Table S1: An example of the construction of *screened*, *unscreened* and *grouped* datasets for calculating the efficacy of Ivermectin (IVM) and Moxidectin (MOX) for a group of 26 animals (dataset GE02)**

| Faecal egg count (epg) | Block | Treatment | Used in efficacy calculation for dataset | | |
| --- | --- | --- | --- | --- | --- |
|  |  |  | *Screened* | *Unscreened* | *Grouped* |
| 187.5 | 1 | MOX | MOX only | MOX only | MOX & IVM |
| 175 | 1 | IVM | IVM only | IVM only | MOX & IVM |
| 150 | 2 | IVM | IVM only | IVM only | MOX & IVM |
| 137.5 | 2 | MOX | MOX only | MOX only | MOX & IVM |
| 87.5 | 3 | MOX | MOX only | MOX only | MOX & IVM |
| 75 | 3 | IVM | IVM only | IVM only | MOX & IVM |
| 75 | 4 | MOX | MOX only | MOX only | MOX & IVM |
| 75 | 4 | IVM | IVM only | IVM only | MOX & IVM |
| 62.5 | 5 | MOX | MOX only | MOX only | MOX & IVM |
| 62.5 | 5 | IVM | IVM only | IVM only | MOX & IVM |
| 62.5 | 6 | IVM | IVM only | IVM only | MOX & IVM |
| 50 | 6 | MOX | MOX only | MOX only | MOX & IVM |
| 50 | 7 | MOX | MOX only | MOX only | MOX & IVM |
| 37.5 | 7 | IVM | IVM only | IVM only | MOX & IVM |
| 37.5 | 8 | MOX | MOX only | MOX only | MOX & IVM |
| 37.5 | 8 | IVM | IVM only | IVM only | MOX & IVM |
| 37.5 | 9 | IVM | IVM only | IVM only | MOX & IVM |
| 37.5 | 9 | MOX | MOX only | MOX only | MOX & IVM |
| 37.5 | 10 | IVM | IVM only | IVM only | MOX & IVM |
| 25 | 10 | MOX | MOX only | MOX only | MOX & IVM |
| 12.5 | 11 | MOX | -- | MOX only | MOX & IVM |
| 12.5 | 11 | IVM | -- | IVM only | MOX & IVM |
| 12.5 | 12 | MOX | -- | MOX only | MOX & IVM |
| 12.5 | 12 | IVM | -- | IVM only | MOX & IVM |
| 0 | 13 | IVM | -- | IVM only | MOX & IVM |
| 0 | 13 | MOX | -- | MOX only | MOX & IVM |

**Table S2: The 27 trials with at least one differing efficacy classification (EFF = efficacious; INC = inconclusive; CAR = confirmed anthelmintic resistance) between Markov chain Monte Carlo and bootstrap methods, or between *screened*, *unscreened* and *grouped* datasets, for moxidectin (MOX) and ivermectin (IVM).**

| Farm | Treatment | *Screened* data | | | *Unscreened* data | | | *Grouped* data | | |
| --- | --- | --- | --- | --- | --- | --- | --- | --- | --- | --- |
|  |  | Pre-N | MCMC | Boot | Pre-N | MCMC | Boot | Pre-N | MCMC | Boot |
| FR04 | MOX | 7 | CAR | INC | 9 | CAR | INC | 21 | CAR | INC |
| FR05 | IVM | 7 | INC | INC | 9 | INC | INC | 19 | INC | INC |
| FR06 | IVM | 9 | CAR | INC | 12 | CAR | INC | 23 | CAR | CAR |
| FR09 | IVM | 10 | INC | EFF | 10 | INC | EFF | 21 | INC | INC |
| GE02 | MOX | 10 | INC | EFF | 13 | INC | INC | 26 | INC | INC |
| GE03 | IVM | 10 | INC | EFF | 10 | INC | EFF | 20 | INC | EFF |
| GE05 | MOX | 10 | EFF | EFF | 13 | INC | INC | 25 | INC | INC |
| GE05 | IVM | 10 | INC | INC | 12 | INC | INC | 25 | INC | EFF |
| GE06 | MOX | 10 | CAR | INC | 13 | CAR | INC | 26 | CAR | CAR |
| GE08 | IVM | 10 | CAR | INC | 11 | CAR | INC | 21 | CAR | INC |
| GE10 | MOX | 10 | INC | EFF | 10 | INC | EFF | 21 | INC | EFF |
| GE12 | MOX | 10 | INC | EFF | 10 | INC | EFF | 20 | INC | INC |
| GE12 | IVM | 10 | CAR | INC | 10 | CAR | INC | 20 | CAR | CAR |
| IT01 | IVM | 9 | CAR | INC | 20 | CAR | INC | 41 | CAR | CAR |
| UK02 | MOX | 10 | INC | INC | 11 | INC | CAR | 22 | CAR | CAR |
| UK07 | MOX | 10 | INC | INC | 25 | INC | INC | 50 | INC | EFF |
| UK07 | IVM | 10 | INC | EFF | 25 | INC | EFF | 50 | INC | EFF |
| UK08 | MOX | 10 | INC | INC | 25 | CAR | CAR | 50 | CAR | CAR |
| UK08 | IVM | 10 | CAR | INC | 25 | CAR | CAR | 50 | CAR | CAR |
| UK10 | MOX | 10 | INC | EFF | 25 | INC | EFF | 50 | INC | EFF |
| UK10 | IVM | 10 | INC | EFF | 25 | INC | EFF | 50 | INC | EFF |
| UK11 | MOX | 9 | INC | EFF | 13 | INC | INC | 27 | INC | INC |
| UK11 | IVM | 9 | INC | INC | 14 | CAR | INC | 27 | CAR | CAR |
| UK12 | IVM | 10 | INC | CAR | 20 | CAR | CAR | 40 | CAR | CAR |
| UK13 | MOX | 10 | INC | EFF | 19 | INC | INC | 37 | INC | INC |
| UK13 | IVM | 9 | CAR | INC | 18 | CAR | INC | 37 | CAR | INC |
| UK14 | MOX | 10 | INC | CAR | 19 | CAR | CAR | 38 | CAR | CAR |
